# Thoracoscopic Diverticulectomy for Epiphrenic Esophageal Diverticulum after Peroral Endoscopic Myotomy: A Report of Four Cases

**DOI:** 10.70352/scrj.cr.25-0065

**Published:** 2025-06-20

**Authors:** Takeshi Yamashita, Koji Otsuka, Masahiro Kohmoto, Akira Saito, Yutaka Kishimoto, Kentaro Motegi, Tomotake Ariyoshi, Satoru Goto, Haruhiro Inoue, Masahiko Murakami, Takeshi Aoki

**Affiliations:** 1 Esophageal Cancer Center, Showa Medical University Hospital, Tokyo, Japan; 2 Division of Gastroenterological and General Surgery, Department of Surgery, School of Medicine, Showa Medical University, Tokyo, Japan; 3 Digestive Diseases Center, Showa Medical University Koto Toyosu Hospital, Tokyo, Japan

**Keywords:** epiphrenic esophageal diverticulum, esophageal achalasia, thoracoscopic esophageal diverticulectomy

## Abstract

**INTRODUCTION:**

An epiphrenic esophageal diverticulum (EED) typically occurs in association with esophageal motility disorders such as esophageal achalasia. Although peroral esophageal myotomy (POEM) is the current standard treatment for esophageal achalasia, laparoscopic diverticulectomy with esophageal myotomy and fundoplication are usually performed for EED. Here, we report four cases of thoracoscopic esophageal diverticulectomy performed for symptomatic EED after POEM for esophageal achalasia.

**CASE PRESENTATION:**

Between 2022 and March 2024, four patients with EED (average diameter, 68 mm) underwent endoscopic esophageal cleaning prior to surgery. POEM was initially performed in all four cases; however, two patients experienced persistent symptoms, while two experienced progressive EED enlargement over the years, necessitating additional surgery. All operations were performed thoracoscopically with the patient in the left lateral position. After resection of the EED, the mediastinal pleura was sutured. Endoscopy, using an endoscopic balloon, was effective in preventing esophageal strictures. No postoperative complications occurred, and the mean postoperative hospital stay was 5.5 (4–8) days. All patients improved postoperatively and remained relapse-free.

**CONCLUSIONS:**

Thoracoscopic esophageal diverticulectomy for large EED can be safely performed with better working space than laparoscopic procedures. Therefore, this technique should be considered a minimally invasive treatment for symptomatic EED cases unresponsive to POEM.

## Abbreviations

EED
: epiphrenic esophageal diverticulum
LES
: lower esophageal sphincter
POEM
: peroral esophageal myotomy

## INTRODUCTION

An epiphrenic esophageal diverticulum (EED) occurs due to increased internal pressure in the lower esophagus caused by failed coordination between esophageal peristaltic movement and the lower esophageal sphincter (LES). It is suggested to be associated with esophageal motility disorders, such as esophageal achalasia.^1)^ Diverticulectomy for EED is generally performed with myotomy and fundoplication, thoracoscopically and/or laparoscopically.^2–4)^ Peroral esophageal myotomy (POEM), a submucosal tunnel technique 1st described in 2010, has since become one of the standard treatments for esophageal motility disorders.^5)^ In cases of EED accompanied by achalasia, reports of POEM alone being effective in improving symptoms are available.^6,7)^ However, only a few reports of diverticulectomy for EED after POEM exist. In this report, we present four cases in which we could safely perform thoracoscopic esophageal diverticulectomy for EED after POEM for esophageal achalasia.

## CASE PRESENTATION

**Fig. 1:**
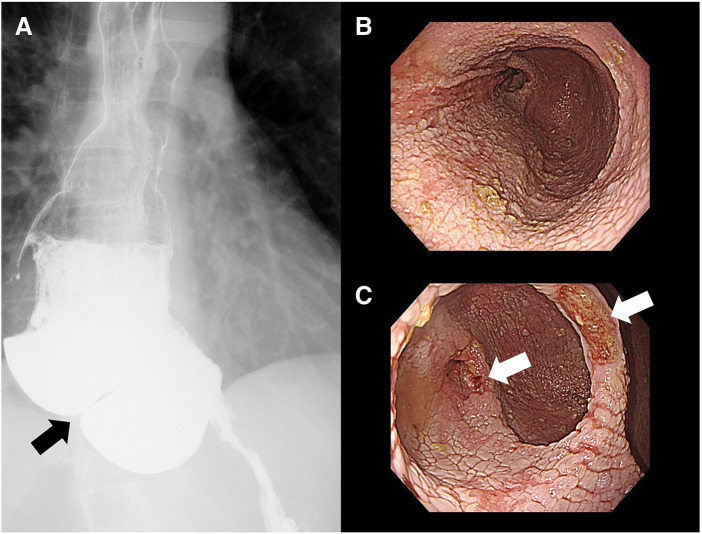
Findings of epiphrenic esophageal diverticulum after peroral esophageal myotomy in patient 4. (**A**) Contrast study using amidotrizoic acid showing the two esophageal diverticula, which are borderline (black arrow). (**B**) Endoscopy image showing the cranial segment of the esophageal diverticulum. (**C**) Endoscopy showing a caudal esophageal diverticulum with ulcerative lesions (white arrow).

**Fig. 2:**
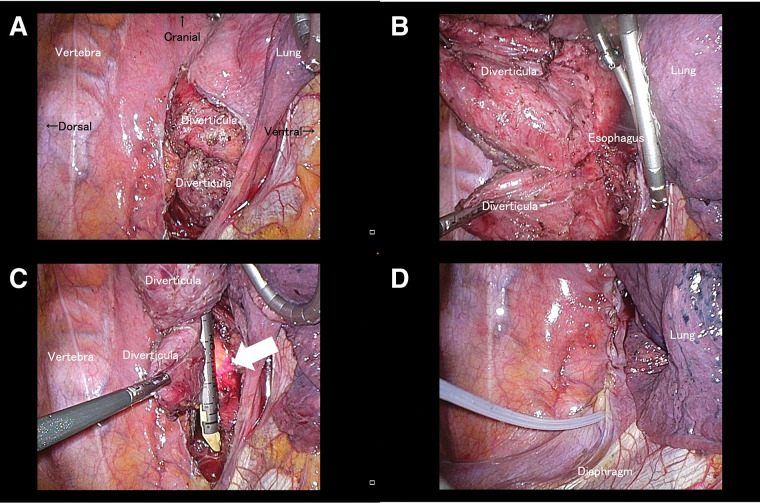
Surgical findings in patient 4. (**A**) Photograph showing the right esophagus after dissection. (**B**) Esophageal diverticula are dissected and exposed. Adhesions are observed on the dorsal and ventral sides of the esophagus. (**C**) Esophageal diverticula are transected using a linear stapler with endoscopic assistance. The light from the endoscope (white arrow) is a landmark of the esophageal wall. (**D**) A 10-Fr silicone drain is inserted into the mediastinal cavity.

**Fig. 3:**
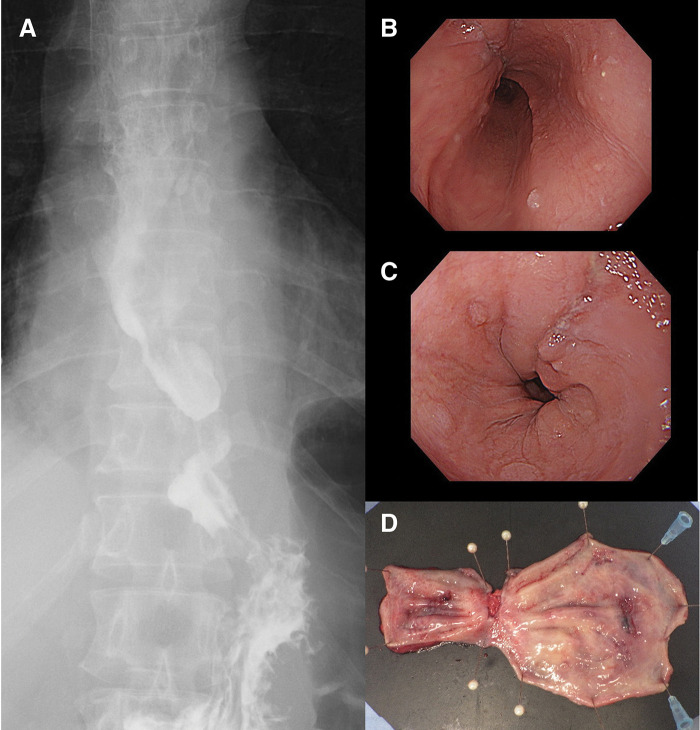
Postoperative findings in patient 4. (**A**) Contrast study using amidotrizoic acid demonstrating that the esophageal diverticula had disappeared. (**B**, **C**) Endoscopy revealing that both esophageal diverticula improved 1 month postoperatively. (**D**) Surgical specimen showing two esophageal diverticula.

We performed thoracoscopic esophageal diverticulectomy in four patients at our hospital between 2022 and March 2024. Computed tomography imaging showed that the EED protruded to the right side of the esophagus in all cases. Although they did not require dietary restrictions, preoperative endoscopic esophageal cleaning was performed in all patients. The mean diverticulum length was 68 mm on contrast examination using amidotrizoic acid. Patients 1 and 2 had no history of treatment for esophageal motility disorders. Although POEM was first performed to improve symptoms, esophageal diverticulectomy was performed because of hematemesis or tarry stools. Patients 3 and 4 had enlarged EED a few years after POEM for esophageal achalasia. Although the patients underwent POEM again, their symptoms, such as dysphagia, did not improve. **Table 1** presents the details of these cases. **Figure 1** shows the findings of the preoperative examinations in patient 4, who interestingly had two diverticula. A thoracoscopic esophageal diverticulectomy was performed with the patients in the left lateral decubitus position under general anesthesia and single-lung ventilation. After five ports were placed in the thoracic wall, the EED was resected using a linear stapler. During the diverticulectomy, an endoscopic balloon was inflated to facilitate resection. Subsequently, the mediastinal pleura was sutured. The mean operative time and blood loss were 162 ± 26 min and 5.5 ± 1 mL, respectively. Meals were introduced 1–3 days postoperatively, provided that a contrast examination using amidotrizoic acid confirmed no leakage or stricture with a smooth passage. The mean (range) postoperative hospital stay was 5.5 (4–8) days, and no postoperative complications occurred. All patients’ symptoms improved postoperatively. **Figures 2** and **3** show the intraoperative findings and postoperative course of patient 4, respectively. Pathological results showed that the EED was a pseudodiverticulum lacking the muscularis propria without malignancy in all cases.

**Table 1:** Characteristics of four cases of thoracoscopic diverticulectomy for epiphrenic esophageal diverticulum after peroral endoscopic myotomy (POEM)

Case	Age (years)	Sex	Duration after the initial poem (months)	Symptoms	Size of diverticula (mm)	Preoperative endoscopic cleaning	Surgery	Surgery time (min)	Bleeding (g)	Contrast study (Days after surgery)	Started meal (Days after surgery)	Postoperative hospital stay (Days)
1	59	Male	5	Hematemesis	68	Yes	Thoracoscopic diverticulectomy	139	7	1	2	5
2	63	Female	4	Vomiting	70	Yes	Thoracoscopic diverticulectomy	145	5	1	1	4
3	49	Male	91	Hematemesis	67	Yes	Thoracoscopic diverticulectomy	167	5	1	1	5
4	49	Male	120	Dysphagia	68, 45	Yes	Thoracoscopic diverticulectomy	197	5	3	3	8

## DISCUSSION

EED, a rare condition occurring in the lower third of the esophagus, is classified as either acquired or pulsion-type.^1)^ Surgical intervention is indicated when EED becomes symptomatic. As EED is often associated with esophageal motility disorders—such as impaired coordination between the distal esophagus and the LES^1)^—diverticulectomy is typically performed in conjunction with myotomy and fundoplication to prevent reflux. Laparoscopic diverticulectomy is commonly employed, as it allows for simultaneous myotomy and fundoplication while offering superior visibility and operability than laparotomy.^8)^ In a review of 133 cases conducted by Hirano et al. (1995–2008), myotomy and fundoplication were performed in 74% of cases, and 84% of the procedures utilized a laparoscopic approach.^4)^ However, anastomotic leakage remains a significant complication, occurring in 15% of cases.^4)^ We believe one contributing factor to this complication is the narrow working space inherent in the transhiatal approach. By contrast, the thoracoscopic approach provides a broader operative field, reducing restrictions on stapling and other maneuvers. In our series, we selected the thoracoscopic approach based on our institutional experience with thoracoscopic esophageal cancer surgery performed in the left lateral decubitus position.^9)^ All surgeries were completed without complications. Sato et al. have proposed a decision-making flowchart supporting the thoracoscopic approach, especially in cases with suspected adhesions, when the inferior margin of the diverticulum neck is ≥5 cm above the esophagogastric junction, when diverticulum size is ≥6 cm, or when food retention is observed.^10)^ In our four cases, the diverticulum size was ≥5 cm. The exact distance from the esophagogastric junction was unclear because of prior POEM procedures, but based on our outcomes, we support the use of the aforementioned flowchart.

In all four cases, POEM was initially performed based on the diagnosis of esophageal achalasia. POEM has emerged as a standard treatment for achalasia.^5)^ Randomized controlled trials have demonstrated that POEM is at least as effective as Heller myotomy and non-inferior to balloon dilation.^11,12)^ Moreover, several reports have described the feasibility of combining POEM with diverticulectomy in patients with achalasia and EED.^6,7)^ Kinoshita et al. reported that in cases of achalasia with an esophageal diverticulum, symptoms are typically driven by achalasia, and POEM alone often improves symptoms, making diverticulectomy unnecessary in some patients.^13)^ A review by Facciorusso et al. similarly suggested that POEM may become the first-line treatment for EED complicated by achalasia.^14)^

In our cohort, patients 1 and 2 underwent diverticulectomy because of persistent symptoms (vomiting, hematemesis) caused by the diverticulum. In patients 3 and 4, the size of the diverticulum increased following POEM, leading to symptom onset and the need for surgical resection. In patient 4, the diverticulum appeared to cause food stasis, leading to esophageal ulceration and reflux esophagitis. Chen et al. reported that 11% of patients undergoing Heller myotomy with fundoplication developed diverticula at the myotomy site, as observed via endoscopy.^15)^ Conversely, Kinoshita et al. reported a trend—albeit not statistically significant—toward a reduction in diverticulum size after POEM.^13)^ We speculate that the observed post-POEM enlargement of diverticula may be because of the esophageal wall following myotomy, thereby promoting diverticular expansion. Continued follow-up of patients after POEM is necessary to verify this hypothesis.

As POEM becomes increasingly adopted as the standard treatment for achalasia, similar cases are likely to become more common. In patients with a history of POEM, where additional myotomy is not required, we propose that the thoracoscopic approach is particularly useful for resecting large esophageal diverticula, as demonstrated in this study. Nonetheless, this management strategy has a weakness: when diverticulectomy is performed after POEM, a second surgery under general anesthesia is necessary, unlike in combined procedures where myotomy and fundoplication are performed simultaneously. Therefore, future studies should compare outcomes between post-POEM diverticulectomy and traditional diverticulectomy with concurrent myotomy.

## CONCLUSIONS

Thoracoscopic esophageal diverticulectomy can be safely performed with better working space than laparoscopic procedures. For symptomatic EED cases unresponsive to POEM, this technique should be considered as minimally invasive treatment. In the future, comparing this procedure with diverticulectomy combined with myotomy and fundoplication in multicenter clinical trials would be beneficial.

## ACKNOWLEDGMENTS

We would like to thank Editage (www.editage.com) for English-language editing.

## DECLARATIONS

### Funding

No financial support was provided.

### Authors’ contributions

TY and KO created the concept and design.

MK, AS, YK, KM, and TA collected the data and contributed to perioperative management.

TY wrote the manuscript.

SG, HI, MM, and TA supervised the study.

All authors have reviewed and approved the final manuscript, and each author agrees to be held accountable for all aspects of the research.

### Availability of data and materials

Data sharing does not apply to this article.

### Ethics approval and consent to participate

This work does not require ethical considerations or approval. Informed consent to participate in this study was obtained from the patient.

### Consent for publication

Written informed consent was obtained from the patient for the publication of this case report.

### Competing interests

The authors have no conflicts of interest to declare.
